# Gender-Affirming Surgical History, Satisfaction, and Unmet Needs Among Transgender Adults

**DOI:** 10.1001/jamanetworkopen.2025.32494

**Published:** 2025-09-18

**Authors:** David R. Pletta, Meg Quint, Asa E. Radix, Kenneth H. Mayer, Jennifer Potter, Devin Coon, Alex S. Keuroghlian, Jaclyn M. W. Hughto, Sari L. Reisner

**Affiliations:** 1 Department of Epidemiology, University of Michigan School of Public Health, Ann Arbor; 2 The Fenway Institute, Fenway Health, Boston, Massachusetts; 3 currently a medical student at Stanford University School of Medicine, Stanford, California; 4 Callen-Lorde Community Health Center, New York, New York; 5 Department of Epidemiology, Columbia University Mailman School of Public Health, New York, New York; 6 Department of Global Health and Population, Harvard T. H. Chan School of Public Health, Boston, Massachusetts; 7 Department of Medicine, Harvard Medical School, Boston, Massachusetts; 8 Center for Transgender Health, Brigham and Women’s Hospital, Boston, Massachusetts; 9 Department of Surgery, Harvard Medical School, Boston, Massachusetts; 10 Department of Psychiatry, Harvard Medical School, Boston, Massachusetts; 11 Department of Behavioral and Social Sciences, Brown University School of Public Health, Providence, Rhode Island; 12 Department of Epidemiology, Brown University School of Public Health, Providence, Rhode Island

## Abstract

**Importance:**

Gender-affirming surgery (GAS) is an effective treatment for gender dysphoria among transgender, nonbinary, and gender diverse (TGD) individuals. Research is needed to assess GAS history, factors associated with GAS satisfaction, desired yet unobtained GAS, and barriers and facilitators to GAS access for TGD individuals.

**Objective:**

To assess of the prevalence of GAS, surgical satisfaction, and encountered barriers among TGD adults and the factors associated with these outcomes.

**Design, Setting, and Participants:**

This cross-sectional study, conducted from October 2024 to June 2025, used baseline electronic survey data from TGD adult primary care patients enrolled in LEGACY, a longitudinal cohort study. Data were collected from February 2019 to March 2021.

**Exposures:**

Age, gender identity, race, Latine or Hispanic ethnicity, educational attainment, homelessness, income, health insurance, HIV status, gender marker change, gender-affirming hormone use, severe psychological distress, self-rated health, and participation during the COVID-19 pandemic.

**Main Outcomes and Measures:**

The main outcomes were receipt of GAS, high surgical satisfaction, and encountered barriers to GAS (eg, waiting lists, cost). Descriptive analyses and logistic regression models, stratified by gender identity, were used to examine the association between the exposures and these outcomes.

**Results:**

The study cohort of 2176 patients were a mean (SD) age of 30.3 (10.3) years; 1413 (64.9%) identified as transmasculine (TM), 763 (35.1%) as transfeminine (TF), and 672 (30.9%) as nonbinary. A total of 634 patients (29.1%) identified as a member of a racial minority group and 237 (10.9%) as Latine or Hispanic. Overall, 946 patients (43.5%) had received GAS, and 776 of these patients (82.0%) reported high satisfaction. The most desired surgeries were hysterectomy (868 of 1413 [61.4%]) for TM patients and facial feminization (516 of 763 [67.6%]) for TF patients. Most patients (2054 [94.4%]) encountered a barrier to GAS, with the most common being cost (1455 [66.9%]). In multivariable models, younger age (eg, 18-24 years vs ≥40 years) was associated with lower odds of GAS (TM patients: adjusted odds ratio [AOR], 0.19 [95% CI, 0.11-0.34]; TF patients: AOR, 0.22 [95% CI, 0.12-0.42]) and higher odds of encountering a barrier (TM patients: AOR, 3.16 [95% CI, 1.59-6.30]; TF patients: AOR, 9.39 [95% CI, 2.47-35.67]). Gender marker change (TM patients: AOR, 8.61 [95% CI, 6.19-11.98]; TF patients: AOR, 6.29 [95% CI, 4.01-9.87) and hormone use (TM patients: AOR, 4.71 [95% CI, 3.02-7.34]; TF patients: AOR, 7.69 [95% CI, 1.79-33.04]) were associated with greater odds of GAS; lack of insurance was associated with lower satisfaction (TM patients: AOR, 0.31 [95% CI, 0.13-0.76]; TF patients: AOR, 0.09 [95% CI, 0.02-0.49]).

**Conclusions and Relevance:**

In this cross-sectional study, TGD patients reported high GAS satisfaction but substantial unmet need and frequent barriers to care. Efforts appear to be needed to improve accessibility for TGD patients desiring GAS.

## Key Points

**Question:**

What is the prevalence of gender-affirming surgery (GAS), high surgical satisfaction, and barriers to GAS among transgender, nonbinary, and gender-diverse (TGD) adults, and what factors are associated with these outcomes?

**Findings:**

In this cross-sectional study of 2176 TGD adult primary care patients, 946 (44%) reported having GAS, 776 of these (82.0%) reported high surgical satisfaction, and 2073 participants (94.4%) encountered a barrier to GAS. GAS prevalence, satisfaction, and barriers differed between transmasculine and transfeminine patients.

**Meaning:**

These findings suggest that there is an unmet need for GAS among TGD patients, warranting interventions to improve the availability and accessibility of these services.

## Teaser

This cross-sectional study assesses the prevalence of and factors associated with gender-affirming surgery (GAS), high surgical satisfaction, and barriers to GAS among transgender, nonbinary, and gender-diverse adults.

## Introduction

In the US, an estimated 1.6 million people are transgender, nonbinary, or gender diverse (TGD), with gender identities varying from those stereotypically ascribed to their sex assigned at birth.^1^ Stemming from this incongruence and exposure to societal stigma surrounding their gender identity, some TGD individuals experience a unique form of psychological distress known as gender dysphoria.^2^ Treatment for gender dysphoria is highly contingent on the needs and desires of TGD individuals and can include psychosocial, hormonal, and surgical interventions.^2^ Gender-affirming surgery (GAS) enables individuals to align their physical bodies more closely with their self-perception, helping alleviate gender dysphoria and improve psychological well-being.^2^ For transfeminine people, GAS may include breast augmentation, body contouring, facial feminization, removal of reproductive organs, and construction of a neovagina; for transmasculine people, GAS may include mastectomy, chest or breast reduction, removal of reproductive organs, and phalloplasty.^3,4^ Major medical organizations, including the World Professional Association for Transgender Health (WPATH), the American Medical Association, and the American College of Physicians,^2,5^ have asserted the medical necessity of gender-affirming care, and multiple studies have demonstrated the psychological benefits of GAS,^6,7^ including reduction of past–30-day psychological distress and past-year suicidal ideation.^8^

Given the positive mental health effects and medical necessity of GAS, access to GAS is vital for TGD people who meet indications for and desire such treatment. However, many TGD people who desire surgery are unable to access the procedures they require.^9^ Of the 92 329 TGD respondents to the 2022 US Transgender Survey, approximately 1 in 4 (26%) experienced an issue with their insurance company within the past year, such as being denied GAS coverage.^10^ Moreover, insurance coverage varies greatly, and individual providers may not accept public insurance, making GAS inaccessible to patients with these types of insurance plans. However, few studies have evaluated GAS access, unmet needs, and satisfaction in primary care patients, preventing clinicians from better facilitating conversations about GAS, improving referral processes, and advocating for reduced GAS barriers. This is of special importance as gender-affirming care is increasingly provided in primary care settings.^2,11^

Prior studies examining GAS satisfaction in TGD people^12,13,14,15,16^ generally indicated high satisfaction and improved mental health outcomes^14^ and low rates of surgical regret.^17,18^ However, many such studies were limited by small sample sizes, insufficient data collection in surgical settings, or noninclusion of contextual factors that may influence surgical satisfaction (eg, housing status, HIV serostatus) or were otherwise considered to provide low-quality evidence.^12,13^ Additional research is needed to replicate and further substantiate GAS satisfaction findings, particularly in primary care, where referrals to GAS are often initiated. This study sought to describe GAS history and desire for GAS, evaluate factors associated with GAS satisfaction, and characterize experienced barriers to GAS in a sample of adult TGD patients from community health centers.

## Methods

### Participants and Procedures

Data for this cross-sectional study came from LEGACY, a longitudinal cohort of TGD adult primary care patients at Fenway Health in Boston, Massachusetts, and Callen-Lorde Community Health in New York City, New York, 2 community health centers specializing in sexual and gender minority health care.^19^ Eligible patients were 18 years or older, were TGD (self-reported at registration or by *International Statistical Classification of Diseases and Related Health Problems, Tenth Revision* codes F64.0-64.9), had a medical visit in the past 12 months, and had a signed patient consent form on file with no research exclusion documented in their medical record. LEGACY methods and full eligibility criteria are published elsewhere.^19^ This analysis used data from participants who provided informed consent to the study, completed a baseline electronic survey in REDCap between February 2019 and March 2021, and provided self-reported gender identity data. All study procedures were approved by the Fenway Health Institutional Review Board. Study design, analysis, results, and limitations are presented in accordance with the Strengthening the Reporting of Observational Studies in Epidemiology (STROBE) reporting guideline.

LEGACY was codesigned with TGD patients and community members. Focus group discussions with patients and ongoing input from the community advisory board (CAB) ensured that the study was conducted with and not on TGD people.^20,21^ For example, the survey incorporated extensive feedback from TGD communities, including the need to reduce the length of the tool and surgical satisfaction questions. In addition, a scientific advisory board (SAB) was established to gather input from clinicians, researchers, and other key stakeholders.

### Measures

#### Demographics

Self-reported demographic variables included nonbinary gender identity (yes or no), gender identity (transgender man, transgender woman, nonbinary assigned female at birth [AFAB], or nonbinary assigned male at birth [AMAB]), age in years (18-24, 25-29, 30-39, or ≥40), select-all-that-apply racial identity (Asian, Black or African American, Latine or Hispanic, White, multiracial, or other [including American Indian or Alaska Native, Native Hawaiian or other Pacific Islander, or another written-in racial identity], collapsed to improve cell sizes for the purpose of descriptive statistics and statistical modeling), Latine or Hispanic ethnicity (yes or no), attainment of at least a 4-year college degree (yes or no), lifetime homelessness (yes or no), individual monthly income ($0-$999, $1000-3999, or≥$4000), health insurance type (none, public, or private), HIV-positive serostatus (yes or no), any gender marker change (eg, changed gender marker on a passport, driver’s license or state identification card, health insurance card, birth certificate, or social security card [yes or no]), and any lifetime use of gender-affirming hormone therapy (yes or no). Racial and ethnic identity were measured in this study due to their potential to confound the association between some of our measured exposures and GAS-related outcomes of interest. Patients who self-reported more than 1 racial identity were recoded as multiracial. For statistical models, racial identity was recoded to White compared with racial minority identity (yes or no) due to sparse data across groups. Gender identity was recoded to transmasculine (transgender man and nonbinary AFAB) or transfeminine (transgender woman and nonbinary AMAB) since different GAS procedures exist for these groups.

#### Psychological Distress Within the Past 2 Weeks

Current psychological distress was assessed using the validated 4-Item Patient Health Questionnaire (PHQ-4).^22^ Participants were asked, “Over the last 2 weeks, how often have you been bothered by the following problems?” Response options were not at all (0), several days (1), less than half the days (2), and nearly every day (3). PHQ-4 scores ranged from 0 to 12 and were categorized as normal (0-2), mild (3-5), moderate (6-8), and severe (9-12).^22^ A dichotomous variable was coded to indicate the presence of clinically significant psychological distress (9-12) vs normal, mild, or moderate symptoms (0-8).

#### Health-Related Quality of Life Self-Rated General Health

A single item from the validated 12-Item Short Form Health Survey (SF-12) assessed self-rated general health: “In general, would you say your health is…?” Responses options were excellent, very good, good, fair, and poor.^23^ A dichotomous variable was created for excellent or very good general health vs good, fair, or poor general health.

#### COVID-19 Pandemic

A variable was created to reflect whether patients completed their baseline survey during the COVID-19 pandemic. This was operationalized as survey completion on or after March 1, 2020, the date the COVID-19 pandemic was declared an emergency in the US.^24^

#### GAS

Measures of GAS were adapted from 2 national surveys of TGD people: the 2015 US Transgender Survey^8^ and the US Transgender Population Survey.^25^ Participants were asked about GAS experiences: age of first seeking and receiving services, surgical history, desires for surgery and unmet needs, and barriers encountered. Since patients could have received or desired multiple and overlapping GAS procedures, surgical history was assessed via a check-all-that-apply question and coded into 5 anatomic regions: head (eg, facial feminization), chest (eg, breast augmentation, mastectomy), abdomen (eg, buttocks augmentation, feminizing or masculinizing body contouring), reproductive (eg, removal of cervix, uterus, 1 or both ovaries, vagina, and testes), and genital (eg, vaginoplasty, labiaplasty, metoidioplasty, phalloplasty, and scrotoplasty). A dichotomous variable was coded to compare participants who reported 1 or more surgical procedures (ie, any GAS) vs none. For each GAS received, participants reported their satisfaction with the procedure. Response options were on a 5-item Likert scale from very dissatisfied (1) to very satisfied (5). Mean patient satisfaction scores were calculated across surgeries, and high surgical satisfaction was coded as a mean score of 4 or greater, aligned with a rating of very satisfied or mostly satisfied.

Following the Consensus-Based Standards for the Selection of Health Measurement Instruments guidelines, content validity of GAS measures was assessed by asking patients, clinicians, and key stakeholders in the CAB and SAB about the comprehensibility of instructions, items, and response options.^26,27^ Measure brevity was prioritized to minimize respondent burden and achieve parent study aims.^19^

### Statistical Analysis

Data analyses were conducted from October 2024 to March 2025, using SAS OnDemand for Academics, version 3.82 (SAS Institute Inc),^28^ and figures were created using R, version 4.4.3 and RStudio, version 2024.12.1 (R Project for Statistical Computing). Descriptive statistics were used to characterize the sample and outcomes of interest, including receipt of GAS, GAS satisfaction, desired GAS, unmet needs for GAS, and barriers to GAS. We examined the distribution of each of these variables by age, gender identity, race, ethnicity, and HIV serostatus. Missingness within variables ranged from 0% (12-Item Short Form Health Survey) to 13.9% (monthly individual income). Bivariate and multivariable logistic regression models were fit to examine the association between our exposures and 3 outcomes: (1) receipt of any GAS, (2) high surgical satisfaction, and (3) encounter of a barrier to GAS. For each outcome, variables demonstrating a statistically significant bivariate association with the outcome were incorporated into a multivariable model. Statistical significance was established at 2-sided *P* = .05. Models were stratified by transmasculine and transfeminine gender identity.

## Results

### Demographic Characteristics of the Sample

Table 1 presents descriptive statistics for the sample overall (N = 2176) and stratified by gender identity. The sample was a mean (SD) age of 30.3 (10.3) years (median age, 28 [IQR, 23-34] years) and was composed of diverse gender identities: 876 transgender men (40.3%), 628 transgender women (28.9%), 537 nonbinary AFAB (24.7%), and 135 nonbinary AMAB (6.2%). Of the total 2176 patients, 1413 (64.9%) identified as transmasculine and 763 (35.1%) as transfeminine. Roughly one-third of the sample (634 [29.1%]) had a racial minority identity, most commonly multiracial (315 [14.5%]) followed by Latine or Hispanic (120 [5.5%]), Black or African American (97 [4.5%]), and Asian (69 [3.2%]); 1526 (70.1%) were White, and 33 (1.5%) were other race. A total of 237 participants (10.9%) identified as Latine or Hispanic ethnicity and 1923 (88.4%) as not Latine or Hispanic. Nearly half (1015 [46.6%]) had a gender marker change, and 1767 (81.2%) used gender-affirming hormones.

**Table 1.** Sample Descriptive Statistics Stratified by Gender Identity

Characteristic	Patients^a^					Test statistic (*df*)^b^	*P* value^c^
	All (N = 2176)	Transgender man (n = 876)	Transgender woman (n = 628)	Nonbinary AFAB (n = 537)	Nonbinary AMAB (n = 135)		
Age, y							
Mean (SD)	30.3 (10.3)	29.0 (9.1)	33.7 (12.9)	28.4 (7.2)	30.8 (11.2)	53.9 (3)	<.001
Median (IQR)	28 (23-34)	27 (22-33)	30 (24-39)	27 (23-31)	27 (23-34)		
18-24	711 (32.7)	322 (36.8)	168 (26.8)	177 (33.0)	44 (32.6)	82.6 (9)	<.001
25-29	552 (25.4)	219 (25.0)	125 (19.9)	171 (31.8)	37 (27.4)		
30-39	593 (27.3)	232 (26.5)	185 (29.5)	144 (26.8)	32 (23.7)		
≥40	316 (14.5)	103 (11.8)	147 (23.4)	45 (8.4)	21 (15.6)		
Missing	4 (0.2)	0	3 (0.5)	0	1 (0.7)		
Racial identity							
Asian	69 (3.2)	25 (2.9)	25 (4.0)	12 (2.2)	7 (5.2)	22.5 (15)	.09
Black or African American	97 (4.5)	38 (4.3)	36 (5.7)	18 (3.4)	5 (3.7)		
Latine or Hispanic	120 (5.5)	60 (6.8)	36 (5.7)	19 (3.5)	5 (3.7)		
White	1526 (70.1)	603 (68.8)	442 (70.4)	389 (72.4)	92 (68.1)		
Multiracial	315 (14.5)	128 (14.6)	76 (12.1)	88 (16.4)	23 (17.0)		
Other^d^	33 (1.5)	14 (1.6)	7 (1.1)	9 (1.7)	3 (2.2)		
Missing	16 (0.7)	8 (0.9)	6 (1.0)	2 (0.4)	0		
Latine or Hispanic ethnicity							
Yes	237 (10.9)	107 (12.2)	67 (10.7)	48 (8.9)	15 (11.1)	3.9 (3)	.28
No	1923 (88.4)	761 (86.9)	555 (88.4)	487 (90.7)	120 (88.9)		
Missing	16 (0.7)	8 (0.9)	6 (1.0)	2 (0.4)	0		
4-y College degree							
Yes	1162 (53.4)	430 (49.1)	284 (45.2)	363 (67.6)	85 (63.0)	72.7 (3)	<.001
No	895 (41.1)	393 (44.9)	309 (49.2)	146 (27.2)	47 (34.8)		
Missing	119 (5.5)	53 (6.1)	35 (5.6)	28 (5.2)	3 (2.2)		
Lifetime homelessness							
Yes	609 (28.0)	250 (28.5)	188 (29.9)	138 (25.7)	33 (24.4)	3.3 (3)	.35
No	1545 (71.0)	616 (70.3)	437 (69.6)	391 (72.8)	101 (74.8)		
Missing	22 (1.0)	10 (1.1)	3 (0.5)	8 (1.5)	1 (0.7)		
Individual monthly income, US$							
0-999	680 (31.3)	277 (31.6)	201 (32.0)	153 (28.5)	49 (36.3)	25.7 (6)	<.001
1000-3999	799 (36.7)	319 (36.4)	189 (30.1)	241 (44.9)	50 (37.0)		
≥4000	394 (18.1)	149 (17.0)	137 (21.8)	83 (15.5)	25 (18.5)		
Missing	303 (13.9)	131 (15.0)	101 (16.1)	60 (11.2)	11 (8.1)		
Health insurance type							
None	69 (3.2)	34 (3.9)	20 (3.2)	11 (2.0)	4 (3.0)	23.2 (6)	<.001
Public	585 (26.9)	215 (24.5)	209 (33.3)	126 (23.5)	35 (25.9)		
Private	1459 (67.0)	594 (67.8)	382 (60.8)	388 (72.3)	95 (70.4)		
Missing	63 (2.9)	33 (3.8)	17 (2.7)	12 (2.2)	1 (0.7)		
HIV-positive serostatus							
Yes	29 (1.3)	8 (0.9)	17 (2.7)	0	4 (3.0)	NA	<.001
No	2147 (98.7)	868 (99.1)	611 (97.3)	537 (100)	131 (97.0)		
Gender marker change							
Yes	1015 (46.6)	538 (61.4)	350 (55.7)	95 (17.7)	32 (23.7)	307.1 (3)	<.001
No	1161 (53.4)	338 (38.6)	278 (44.3)	442 (82.3)	103 (76.3)		
Lifetime gender-affirming hormone use							
Yes	1767 (81.2)	801 (91.4)	566 (90.1)	308 (57.4)	92 (68.1)	304.3 (3)	<.001
No	398 (18.3)	72 (8.2)	61 (9.7)	224 (41.7)	41 (30.4)		
Missing	11 (0.5)	3 (0.3)	1 (0.2)	5 (0.9)	2 (1.5)		
Psychological distress symptom level^e^							
Normal	616 (28.3)	297 (33.9)	181 (28.8)	103 (19.2)	35 (25.9)	48.5 (9)	<.001
Mild	702 (32.3)	255 (29.1)	225 (35.8)	177 (33.0)	45 (33.3)		
Moderate	471 (21.6)	188 (21.5)	114 (18.2)	142 (26.4)	27 (20.0)		
Severe	383 (17.6)	134 (15.3)	106 (16.9)	115 (21.4)	28 (20.7)		
Missing	4 (0.2)	2 (0.2)	2 (0.3)	0	0		
Self-reported general health^f^							
Poor	67 (3.1)	16 (1.8)	20 (3.2)	26 (4.8)	5 (3.7)	33.0 (12)	.001
Fair	298 (13.7)	106 (12.1)	82 (13.1)	92 (17.1)	18 (13.3)		
Good	798 (36.7)	331 (37.8)	210 (33.4)	202 (37.6)	55 (40.7)		
Very good	791 (36.4)	338 (38.6)	232 (36.9)	175 (32.6)	46 (34.1)		
Excellent	221 (10.2)	84 (9.6)	84 (13.4)	42 (7.8)	11 (8.1)		
Missing	1 (0.05)	1 (0.1)	0	0	0		
Survey during COVID-19 pandemic							
Yes	2045 (94.0)	812 (92.7)	594 (94.6)	507 (94.4)	132 (97.8)	6.6 (3)	.09
No	131 (6.0)	64 (7.3)	34 (5.4)	30 (5.6)	3 (2.2)		

Abbreviations: AFAB, assigned female at birth; AMAB, assigned male at birth; NA, not available.

^a^ Data are presented as number (percentage) of patients unless otherwise indicated.

^b^ Statistical tests included Kruskal-Wallis tests for continuous variables, Pearson χ^2^ tests of independence for categorical variables where all cell sizes were greater than 5, and Fisher exact tests for categorical variables where cell sizes were less than 5. Missing values were excluded from testing.

^c^ Statistical significance was established at α = .05.

^d^ Includes American Indian or Alaskan Native, Native Hawaiian or Other Pacific Islander, or another written-in racial identity that did not fit within one of the noncollapsed groups.

^e^ Measured using the 4-Item Patient Health Questionnaire. A score of 0 to 2 indicated normal; 3 to 5, mild; 6 to 8, moderate; and 9 to 12, severe psychological distress symptoms.

^f^ Measured using the 12-Item Short Form Health Survey.

### GAS History and Surgical Satisfaction

Table 2 shows GAS prevalence and surgical satisfaction stratified by gender identity. Overall, 946 of 2176 patients (43.5%) had received at least 1 form of GAS, including 511 of 876 transgender men (58.3%), 229 of 628 transgender women (36.5%), 182 of 537 nonbinary AFAB patients (33.9%), and 24 of 135 nonbinary AMAB patients (17.8%). Among these patients, GAS prevalence varied by gender identity, with the most common surgical region being chest for transgender men (490 of 511 [95.9%]), head and neck for transgender women (137 of 229 [59.8%]), chest for nonbinary AFAB patients (165 of 182 [90.7%]), and both head and neck and reproductive surgeries for nonbinary AMAB patients (both 14 of 24 [58.3%]).

**Table 2.** GAS History and Surgical Satisfaction Stratified by Gender Identity

Characteristic	Patients^a^					Test statistic (*df*)^b^	*P* value^c^
	All (N = 2176)	Transgender man (n = 876)	Transgender woman (n = 628)	Nonbinary AFAB (n = 537)	Nonbinary AMAB (n = 135)		
Any GAS	946 (43.5)	511 (58.3)	229 (36.5)	182 (33.9)	24 (17.8)	147.6 (3)	<.001
Age, mean (SD), y							
First sought GAS	26.5 (9.62)	24.9 (8.22)	29.1 (11.3)	24.5 (6.9)	31.0 (13.2)	41.5 (3)	<.001
First received GAS	28.3 (9.7)	26.6 (8.2)	31.2 (11.4)	26.4 (6.7)	31.5 (13.1)	42.7 (3)	<.001
Total reported GAS procedures, No.	1377	690	418	221	48	NA	NA
GAS by anatomic region^d^							
Head and neck	189 (20.0)	25 (4.9)	137 (59.8)	13 (7.1)	14 (58.3)	75.7 (3)	<.001
Chest	733 (77.5)	490 (95.9)	71 (31.0)	165 (90.7)	7 (29.2)	252.4 (3)	<.001
Body contouring	44 (4.7)	19 (3.7)	17 (7.4)	6 (3.3)	2 (8.3)	NA	.003
Reproductive	293 (31.0)	136 (26.6)	107 (46.7)	36 (19.8)	14 (58.3)	15.5 (3)	.002
Genital	118 (12.5)	20 (3.9)	86 (37.6)	1 (0.5)	11 (45.8)	NA	<.001
Surgical satisfaction score, mean (SD)^e^							
Head and neck	3.8 (1.2)	3.6 (1.3)	3.8 (1.0)	4.1 (0.8)	4.1 (1.1)	2.0 (3)	.57
Chest	4.3 (1.0)	4.3 (1.0)	4.1 (1.3)	4.4 (0.9)	3.3 (1.4)	8.2 (3)	.04
Abdomen	3.8 (1.1)	4.1 (1.0)	3.4 (1.0)	3.8 (1.2)	3.0 (NA)	4.0 (3)	.27
Reproductive	4.6 (1.0)	4.6 (0.9)	4.5 (1.1)	4.4 (1.1)	4.5 (1.1)	1.1 (3)	.78
Genital	4.0 (1.2)	3.8 (1.1)	4.0 (1.2)	4.0 (NA)	3.6 (1.5)	1.7 (3)	.65
High surgical satisfaction^f^							
Yes	776 (82.0)	434 (84.9)	168 (73.4)	157 (86.3)	17 (70.8)	18.8 (3)	<.001
No	167 (17.7)	75 (14.7)	60 (26.2)	25 (13.7)	7 (29.2)		
Missing	3 (0.3)	2 (0.4)	1 (0.4)	0	0		

Abbreviations: AFAB, assigned female at birth; AMAB, assigned male at birth; GAS, gender-affirming surgery; NA, not available.

^a^ Data are presented as number (percentage) of patients unless otherwise indicated.

^b^ Statistical tests included Kruskal-Wallis tests for continuous variables and Pearson χ^2^ tests of independence for categorical variables where all cell sizes were greater than 5. Missing values were excluded from testing.

^c^ Statistical significance was established at α = .05.

^d^ Patients selected all that applied. Denominator is total number of patients receiving any GAS.

^e^ Scores range from 1 (very dissatisfied) to 5 (very satisfied).

^f^ Indicates scores of 4 or greater. Denominator is total number of patients receiving any GAS.

Of the 946 patients with prior GAS, 776 (82.0%) reported high surgical satisfaction, as calculated across prior surgeries. While mean satisfaction scores varied by gender identity and anatomic region, reproductive surgeries were consistently rated the most satisfactory across gender identity groups (mean [SD], 4.6 [1.0]; range of means: 4.4 [nonbinary AFAB] to 4.6 [transgender men]). The frequency of specific surgical procedures and mean levels of satisfaction by gender identity are presented in eTable 1 in Supplement 1.

### GAS Procedure Types and Unmet Surgical Needs

The Figure displays the distribution of received and desired surgeries among transmasculine and transfeminine patients, with stratified plots for (1) all transmasculine and transfeminine patients, (2) binary transmasculine and transfeminine patients, and (3) nonbinary transmasculine and transfeminine patients (corresponding data are in eTable 1 in Supplement 1). Among transmasculine patients, the most frequently received procedure was double mastectomy (637 of 1413 [45.1%]), followed by uterus removal (161 of 1413 [11.4%]) and cervix removal (150 of 1413 [10.6%]). The most desired and unmet GAS types were uterus removal (868 of 1413 [61.4%]) followed by oophorectomy (845 of 1413 [59.8%]) and cervix removal (703 of 1413 [49.8%]). Among transfeminine patients, the most common procedure was orchiectomy (118 of 763 [15.5%]) followed by vaginoplasty (95 of 763 [12.5%]) and labiaplasty (82 of 763 [10.7%]). The most desired and unmet GAS type was facial femininization (516 of 763 [67.6%]) followed by labiaplasty (466 of 763 [61.1%]) and vaginoplasty (465 of 763 [60.9%]).

**Figure.**
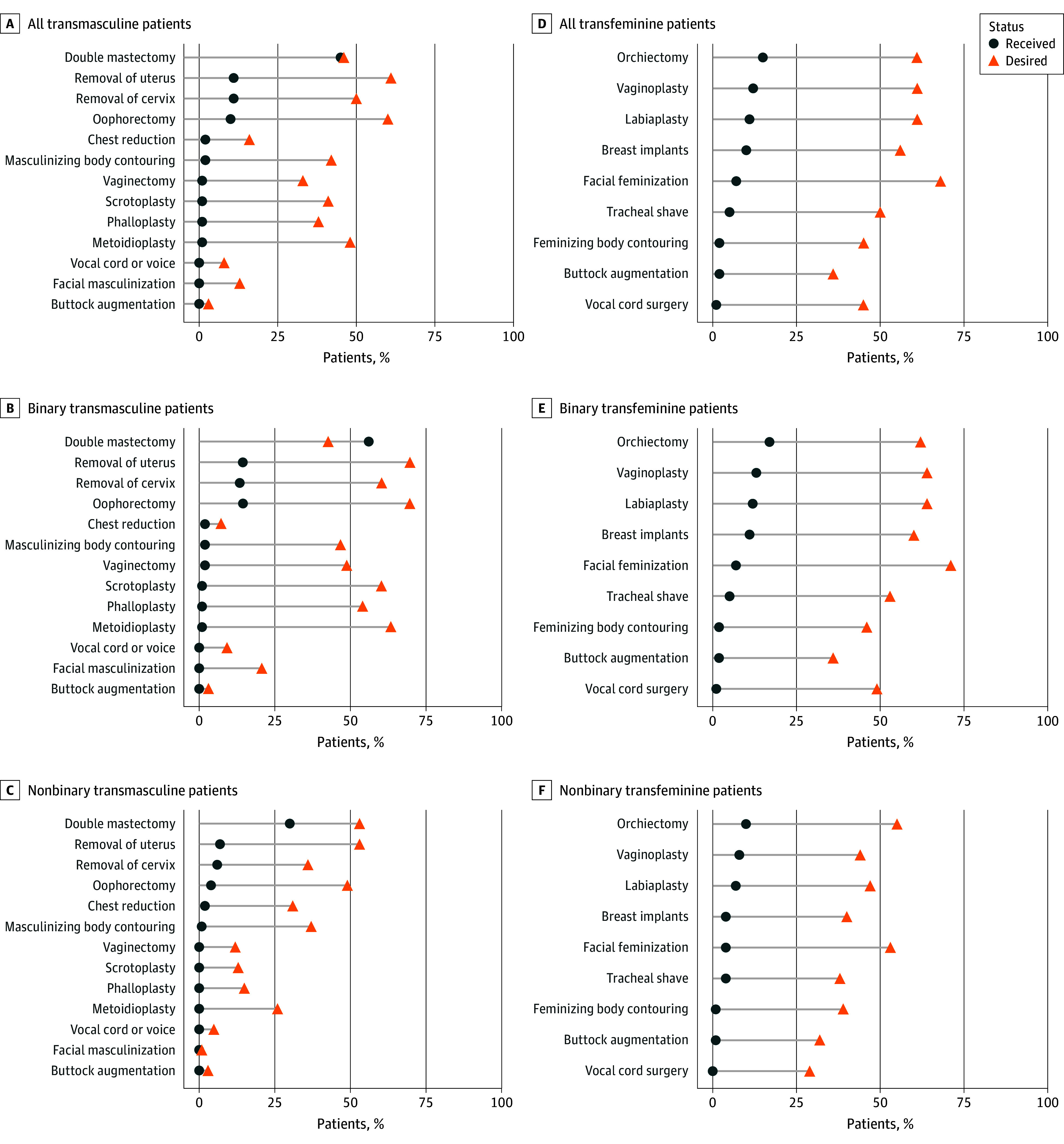
Received and Desired Gender-Affirming Surgeries by Gender Identity

### Barriers to Surgery

eTable 2 in Supplement 1 presents the frequency of patient-reported GAS barriers. Within the total sample, 2054 patients (94.4%) experienced at least 1 barrier to GAS. Frequent themes for barriers included cost, worries about medical complications, concerns about postoperative care, logistical and financial constraints (eg, not being able to work), and lack of clinician availability (eg, long waiting lists, unable to find a transgender-affirming clinician). Multivariable models assessing factors associated with lifetime GAS, high GAS satisfaction, and encountering a barrier to GAS stratified by transmasculine and transfeminine patients are detailed in Table 3 and Table 4, respectively.

**Table 3.** Logistic Regression Results Among Transmasculine Patients^a^

Characteristic	Any GAS (n = 125)		High surgical satisfaction (n = 667)		Barrier to GAS (n = 1330)	
	OR (95% CI)	AOR (95% CI)	OR (95% CI)	AOR (95% CI)	OR (95% CI)	AOR (95% CI)
Gender nonbinary						
Yes	0.37 (0.29-0.46)	1.10 (0.78-1.56)	1.09 (0.67-1.77)	NA	0.78 (0.50-1.22)	NA
No	1 [Reference]	NA	1 [Reference]	NA	1 [Reference]	NA
Age category, y						
18-24	0.19 (0.13-0.29)	0.19 (0.11-0.34)	3.61 (1.71-7.59)	3.49 (1.55-7.89)	3.74 (1.95-7.17)	3.16 (1.59-6.30)
25-29	0.42 (0.28-0.63)	0.37 (0.22-0.65)	2.37 (1.26-4.48)	2.23 (1.13-4.41)	3.91 (1.94-7.86)	4.12 (1.98-8.56)
30-39	0.65 (0.43-0.98)	0.52 (0.30-0.91)	1.33 (0.75-2.33)	1.23 (0.68-2.24)	1.80 (0.99-3.29)	2.05 (1.09-3.84)
≥40	1 [Reference]	1 [Reference]	1 [Reference]	1 [Reference]	1 [Reference]	1 [Reference]
White racial identity						
Yes	1.18 (0.94-1.48)	NA	1.24 (0.78-1.97)	NA	1.18 (0.73-1.89)	NA
No	1 [Reference]	NA	1 [Reference]	NA	1 [Reference]	NA
Latine or Hispanic ethnicity						
Yes	0.84 (0.60-1.17)	NA	0.63 (0.33-1.18)	NA	0.72 (0.38-1.36)	NA
No	1 [Reference]	NA	1 [Reference]	NA	1 [Reference]	NA
4-y College degree						
Yes	2.11 (1.69-2.64)	1.71 (1.21-2.40)	0.80 (0.49-1.30)	NA	0.53 (0.32-0.89)	0.66 (0.38-1.15)
No	1 [Reference]	1 [Reference]	1 [Reference]	NA	1 [Reference]	1 [Reference]
Lifetime homelessness						
Yes	0.95 (0.75-1.21)	NA	0.56 (0.36-0.88)	0.76 (0.46-1.26)	1.04 (0.64-1.71)	NA
No	1 [Reference]	NA	1 [Reference]	1 [Reference]	1 [Reference]	NA
Monthly income, US$						
0-999	1 [Reference]	1 [Reference]	1 [Reference]	NA	1 [Reference]	NA
1000-3999	1.41 (1.09-1.81)	0.95 (0.67-1.35)	0.80 (0.46-1.40)	NA	0.92 (0.53-1.61)	NA
≥4000	2.52 (1.81-3.50)	1.03 (0.64-1.66)	0.82 (0.43-1.54)	NA	0.60 (0.32-1.14)	NA
Health insurance type						
None	1.54 (0.84-2.84)	NA^b^	0.27 (0.11-0.63)	0.31 (0.13-0.76)	2.46 (0.33-18.16)	NA^b^
Public	0.94 (0.74-1.21)	NA^b^	0.78 (0.47-1.29)	0.98 (0.57-1.68)	0.58 (0.36-0.93)	NA^b^
Private	1 [Reference]	NA	1 [Reference]	1 [Reference]	1 [Reference]	NA
HIV-positive serostatus						
Yes	1.74 (0.41-7.30)	NA	0.11 (0.02-0.67)	0.12 (0.02-0.79)	0.45 (0.05-3.66)	NA
No	1 [Reference]	NA	1 [Reference]	1 [Reference]	1 [Reference]	NA
Gender marker change						
Yes	12.63 (9.79-16.30)	8.61 (6.19-11.98)	1.50 (0.95-2.36)	NA	0.78 (0.50-1.21)	NA
No	1 [Reference]	1 [Reference]	1 [Reference]	NA	1 [Reference]	NA
Lifetime GAH use						
Yes	10.14 (7.04-14.60)	4.71 (3.02-7.34)	1.41 (0.60-3.30)	NA	0.69 (0.38-1.27)	NA
No	1 [Reference]	1 [Reference]	1 [Reference]	NA	1 [Reference]	NA
Clinically significant psychological distress^c^						
Yes	0.63 (0.48-0.84)	0.77 (0.51-1.14)	0.86 (0.48-1.54)	NA	2.49 (1.13-5.46)	2.67 (1.06-6.74)
No	1 [Reference]	1 [Reference]	1 [Reference]	NA	1 [Reference]	1 [Reference]
High self-rated health^d^						
Yes	1.32 (1.07-1.63)	1.12 (0.83-1.51)	0.91 (0.59-1.39)	NA	0.72 (0.46-1.12)	NA
No	1 [Reference]	1 [Reference]	1 [Reference]	NA	1 [Reference]	NA
During COVID-19 pandemic						
Yes	0.64 (0.46-0.79)	0.73 (0.51-1.04)	0.87 (0.50-1.52)	NA	1.62 (0.87-3.02)	NA
No	1 [Reference]	1 [Reference]	1 [Reference]	NA	1 [Reference]	NA

Abbreviations: AOR, adjusted odds ratio; GAH, gender-affirming hormone; GAS, gender-affirming surgery; NA, not applicable.

^a^ Variables with a significant bivariate association with the outcome (ORs) were incorporated into a multivariable model (AORs).

^b^ The covariate resulted in a positivity violation and was dropped.

^c^ A 4-Item Patient Health Questionnaire score of 9 or more points.

^d^ From the general health item of the 12-Item Short Form Health Survey.

**Table 4.** Logistic Regression Results Among Transfeminine Patients^a^

Characteristic	Any GAS (n = 641)		High surgical satisfaction (n = 249)		Barrier to GAS (n = 718)	
	OR (95% CI)	AOR (95% CI)	OR (95% CI)	AOR (95% CI)	OR (95% CI)	AOR (95% CI)
Gender nonbinary						
Yes	0.38 (0.24-0.60)	0.86 (0.47-1.56)	0.87 (0.34-2.19)	NA	0.33 (0.16-0.66)	0.33 (0.15-0.76)
No	1 [Reference]	NA	1 [Reference]	NA	1 [Reference]	NA
Age category, y						
18-24	0.13 (0.08-0.21)	0.22 (0.12-0.42)	1.13 (0.45-2.86)	NA	7.84 (2.26-27.24)	9.39 (2.47-35.67)
25-29	0.40 (0.26-0.63)	0.51 (0.29-0.90)	1.47 (0.67-3.21)	NA	2.49 (1.01-6.18)	4.61 (1.61-13.17)
30-39	0.44 (0.29-0.67)	0.36 (0.22-0.62)	1.32 (0.67-2.60)	NA	2.33 (1.04-5.23)	3.72 (1.44-9.60)
≥40	1 [Reference]	1 [Reference]	1 [Reference]	NA	1 [Reference]	1 [Reference]
White racial identity						
Yes	1.19 (0.85-1.66)	NA	1.36 (0.74-2.52)	NA	1.38 (0.68-2.77)	NA
No	1 [Reference]	NA	1 [Reference]	NA	1 [Reference]	NA
Latine or Hispanic ethnicity						
Yes	0.71 (0.43-1.19)	NA	0.75 (0.29-1.92)	NA	2.12 (0.50-9.00)	NA
No	1 [Reference]	NA	1 [Reference]	NA	1 [Reference]	NA
4-y College degree						
Yes	1.57 (1.15-2.14)	1.10 (0.72-1.71)	1.02 (0.58-1.81)	NA	0.30 (0.14-0.68)	0.46 (0.20-1.07)
No	1 [Reference]	1 [Reference]	1 [Reference]	NA	1 [Reference]	1 [Reference]
Lifetime homelessness						
Yes	1.07 (0.77-1.49)	NA	0.82 (0.45-1.50)	NA	0.66 (0.33-1.31)	NA
No	1 [Reference]	NA	1 [Reference]	NA	1 [Reference]	NA
Monthly income, US$						
0-999	1 [Reference]	1 [Reference]	1 [Reference]	NA	1 [Reference]	NA
1000-3999	1.11 (0.75-1.65)	0.89 (0.55-1.44)	0.59 (0.27-1.26)	NA	1.07 (0.43-2.67)	NA
≥4000	2.80 (1.85-4.24)	1.64 (0.94-2.84)	0.91 (0.42-1.96)	NA	1.08 (0.39-3.04)	NA
Health insurance type						
None	1.25 (0.53-2.91)	NA	0.08 (0.02-0.40)	0.09 (0.02-0.49)	1.06 (0.14-8.22)	NA
Public	1.13 (0.82-1.57)	NA	0.64 (0.35-1.16)	0.59 (0.32-1.10)	0.70 (0.36-1.39)	NA
Private	1 [Reference]	NA	1 [Reference]	1 [Reference]	1 [Reference]	NA
HIV-positive serostatus						
Yes	0.62 (0.23-1.72)	NA	NA^b^	NA	NA^b^	NA
No	1 [Reference]	NA	NA	NA	NA	NA
Gender marker change						
Yes	9.25 (6.37-13.44)	6.29 (4.01-9.87)	2.66 (1.34-5.26)	2.65 (1.27-5.53)	0.53 (0.26-1.05)	NA
No	1 [Reference]	1 [Reference]	1 [Reference]	1 [Reference]	1 [Reference]	NA
Lifetime GAH use						
Yes	30.82 (7.54-125.99)	7.69 (1.79-33.04)	2.79 (0.17-45.16)	NA	2.35 (1.07-5.18)	2.88 (1.13-7.31)
No	1 [Reference]	1 [Reference]	1 [Reference]	NA	1 [Reference]	1 [Reference]
Clinically significant psychological distress^c^						
Yes	0.61 (0.40-0.93)	0.69 (0.40-1.19)	0.97 (0.42-2.20)	NA	1.39 (0.53-3.63)	NA
No	1 [Reference]	1 [Reference]	1 [Reference]	NA	1 [Reference]	NA
High self-rated health^d^						
Yes	1.47 (1.09-1.99)	0.97 (0.65-1.46)	2.27 (1.29-4.02)	2.21 (1.22-4.02)	0.50 (0.25-1.00)	NA
No	1 [Reference]	1 [Reference]	1 [Reference]	1 [Reference]	1 [Reference]	NA
During COVID-19 pandemic						
Yes	0.74 (0.51-1.07)	NA	0.57 (0.29-1.10)	NA	0.54 (0.27-1.08)	NA
No	1 [Reference]	NA	1 [Reference]	NA	1 [Reference]	NA

Abbreviations: AOR, adjusted odds ratio; GAH, gender-affirming hormone; GAS, gender-affirming surgery; NA, not applicable.

^a^ Variables with a significant bivariate association with the outcome (ORs) were incorporated into a multivariable model (AORs).

^b^ The covariate resulted in a positivity violation, and no estimates were produced.

^c^ Defined as a 4-Item Patient Health Questionnaire score of 9 or more points.

^d^ From the general health item of the 12-Item Short Form Health Survey.

### Transmasculine Patients

Variables associated with higher odds of lifetime GAS for transmasculine patients were younger age (eg, 18-24 years vs ≥40 years: adjusted odds ratio [AOR], 0.19 [95% CI, 0.11-0.34]), 4-year degree (AOR, 1.71 [95% CI, 1.21-2.40]), gender marker change (AOR, 8.61 [95% CI, 6.19-11.98]), and any lifetime use of gender-affirming hormone therapy (AOR, 4.71 [95% CI, 3.02-7.34]). Younger transmasculine patients had higher odds of high surgical satisfaction compared with patients older than 40 years (18-24 years: AOR, 3.49 [95% CI, 1.55-7.89]; 25-29 years: AOR, 2.23 [95% CI, 1.13-4.41];). Transmasculine patients without insurance vs with private insurance (AOR, 0.31 [95% CI, 0.13-0.76]) and HIV-positive vs HIV-negative serostatus (AOR, 0.12 [95% CI, 0.02-0.79]) had lower odds of high surgical satisfaction. Younger age (eg, 18-24 years vs ≥40 years: AOR, 3.16 [95% CI, 1.59-6.30]) and clinically significant psychological distress (AOR, 2.67 [95% CI, 1.06-6.74]) were associated with higher odds of having encountered a barrier to GAS.

### Transfeminine Patients

Younger transfeminine patients had significantly lower odds of any GAS compared with patients older than 40 years (eg, 18-24 years: AOR, 0.22 [95% CI, 0.12-0.42]). Gender marker change (AOR, 6.29 [95% CI, 4.01-9.87]) and any lifetime hormone therapy use (AOR, 7.69 [95% CI, 1.79-33.04]) were associated with higher odds of GAS.

Transfeminine patients without insurance had significantly lower odds of high surgical satisfaction than those with private insurance (AOR, 0.09 [95% CI, 0.02-0.49]). Gender marker change (AOR, 2.65 [95% CI, 1.27-5.53]) and high vs low self-rated general health (AOR, 2.21 [95% CI, 1.22-4.02]) were associated with high GAS satisfaction.

Nonbinary transfeminine patients had significantly lower odds of encountering a barrier to GAS compared with binary transfeminine patients (AOR, 0.33 [95% CI, 0.15-0.76]). Younger age (eg, 18-24 years vs ≥40 years: AOR, 9.39 [95% CI, 2.47-35.67]) and any lifetime gender-affirming hormone use (AOR, 2.88 [95% CI, 1.13-7.31]) were associated with higher odds of having encountered a barrier to GAS.

## Discussion

In this cross-sectional study of TGD primary care patients, we observed a high desire for GAS and a substantial unmet need across most GAS types. The most desired yet unobtained procedures were removal of the uterus for transmasculine patients and facial feminization for transfeminine patients. Among both transmasculine and transfeminine patients, younger age was associated with significantly lower odds of prior GAS. Most patients (94.4%) reported a barrier to GAS. Common barriers related to structural factors, such as costs and unaffordability, being underinsured or uninsured, and lack of clinician access or availability (eg, long waiting lists, finding gender-affirming clinicians). These findings align with previous investigations reporting barriers, including high out-of-pocket costs, lack of insurance coverage, lack of available trained surgeons, and difficulty finding transgender-knowledgeable surgeons.^29,30,31^ Other patient-reported barriers were individual or interpersonal, relating to worry about complications, postoperative care, finding supportive caregivers, and logistical challenges (eg, taking time off). These barriers are meaningful, as unmet needs have far-reaching social, psychological, and economic consequences for TGD people. For example, legal gender marker changes are sometimes predicated on having received GAS. Efforts are needed within health care systems to systematically evaluate barriers to GAS and develop plans to support TGD patients in overcoming obstacles.

Overall, participants with GAS reported high surgical satisfaction, corroborating a review of GAS outcomes in a variety of small or clinic-based studies.^12,13^ Within the sample in our study, lowest surgical satisfaction scores were reported for abdominal, head and neck, and genital surgeries. Low satisfaction rates may relate to high complication rates for genital and head procedures or discordance between patient expectations and outcomes.^32,33,34,35,36^ In transfeminine patients, high self-rated general health was associated with increased odds of high GAS satisfaction. Both transmasculine and transfeminine individuals without insurance had lower odds of high surgical satisfaction compared with those privately insured. TGD patients without insurance may pay for needed surgeries out of pocket through loans or credit cards; thus, they may opt for the lowest-cost surgeon or procedure or settle for a more limited procedure, which may compromise quality and satisfaction. Furthermore, debt incurred for surgery may adversely affect long-term financial well-being. Since our survey did not assess whether patients had health insurance at the time of GAS, it is unknown whether patients became uninsured after the surgery. Future research may benefit from in-depth exploration of health insurance status and surgical satisfaction.

In addition, both transmasculine and transfeminine patients with prior gender-affirming hormone use had higher odds of having any GAS compared with hormone-naive participants. However, hormone-experienced patients did not have higher odds of high surgical satisfaction. Historically, the WPATH Standards of Care (SOC), version 7; individual physicians; and mental health clinicians writing GAS letters have recommended or required that patients use gender-affirming hormone therapy prior to GAS, particularly for genital surgery.^37^ The current WPATH SOC guidelines, version 8, published since these data were collected, remove hormone recommendations for GAS, which may increase access and reduce gatekeeping practices.^2^ The high surgical satisfaction found in this study, irrespective of gender-affirming hormone use, highlights the relevance of contemporary WPATH SOC guidelines and the importance of tailoring gender-affirming care to TGD individuals’ unique needs.

### Limitations

This study has some limitations. The primary care patient sample was derived from 2 community health centers in Boston and New York City that specialize in sexual and gender minority care. This sample may not be generalizable to other clinical populations. For example, most of the study population was privately insured, White non-Latine or non-Hispanic, and highly educated. This urban sample may have greater geographic access to surgeons capable of performing GAS than rural samples. The sociopolitical climate in the Northeast US is also not representative of the entire country. Studies are needed that engage patients from racially, ethnically, and educationally diverse populations across varied geographic regions. Future research may also benefit from longitudinally studying TGD patients’ experiences, desires, and satisfaction with GAS.

LEGACY was not developed to assess clinical surgical outcomes specifically. The single-item satisfaction question asked for each surgical type was adapted from prior research^8,25^ with input from the CAB, SAB, investigators, and staff to parsimoniously assess GAS receipt and general surgical satisfaction in a primary care setting with low respondent burden. Prior studies on patient satisfaction with GAS procedures have used heterogenous measures, many of which were not validated in TGD populations.^12,13,38^ Future research may benefit from using validated multidimensional instruments and patient-reported outcome measures, such as the GENDER-Q,^39,40^ developed with input from TGD communities.

## Conclusions

This cross-sectional study examined GAS history, satisfaction, and unmet need among TGD primary care patients from community health centers. Findings revealed a high desire and considerable unmet need for most GAS types, with many patients reporting barriers to care. The findings suggest that comprehensive efforts are needed within health care systems to improve access to GAS (eg, improving referral pathways, training and hiring more surgeons competent in performing GAS), as access to GAS can advance social affirmation, feelings of mind-body congruence, and psychological well-being for TGD patients.

## Notes

Supplement 1. **eTable 1.** Frequency of Received and Desired Gender-Affirming Surgery and Mean Procedural Satisfaction Scores, by Gender Identity **eTable 2.** Encountered Barriers to Gender-Affirming Surgery (N = 2176)

Supplement 2.
Data Sharing Statement
