# Supplementary material for: Gender-Affirming Surgical History, Satisfaction, and Unmet Needs Among Transgender Adults
Source: JAMA Netw Open. 2025 Sep 18;8(9):e2532494. doi: 10.1001/jamanetworkopen.2025.32494 (PMC12447237; doi:10.1001/jamanetworkopen.2025.32494)
Supplement: Supplement 1. — eTable 1. Frequency of Received and Desired Gender-Affirming Surgery and Mean Procedural Satisfaction Scores, by Gender Identity eTable 2. Encountered Barriers to Gender-Affirming Surgery (N = 2176) [file jamanetwopen-e2532494-s001.pdf]

Supplementary Online Content

Pletta DR, Quint M, Radix AE, et al. Gender-affirming surgical history, satisfaction, and unmet needs among transgender adults. *JAMA Netw Open.* 2025;8(9):e2532494. doi:10.1001/jamanetworkopen.2025.32494

eTable 1. Frequency of Received and Desired Gender-Affirming Surgery and Mean Procedural Satisfaction Scores, by Gender Identity
eTable 2. Encountered Barriers to Gender-Affirming Surgery (N = 2176)

This supplementary material has been provided by the authors to give readers additional information about their work.

© 2025 Pletta DR et al. *JAMA Network Open*.

eTable 1. Frequency of Received and Desired Gender-Affirming Surgery and Mean Procedural Satisfaction Scores, by Gender Identity

Gender Identity	Surgery	Binary Status	N	Surgery Status: Desired but Unmet, No. (%)	Surgery Status: Received, No. (%)	N with Satisfaction Data, No. (%)	Procedural Satisfaction, Mean (SD)	*H* (df)	p-value
Transmasculine	Butt augmentation	All	1413	43 (3.0)	NA	NA	NA	NA	NA
		Binary	876	25 (2.9)	NA	NA	NA		
		Nonbinary	537	18 (3.4)	NA	NA	NA		
	Chest reduction	All	1413	231 (16.3)	27 (1.9)	25 (92.6)	3.1 (1.2)	1.0 (1)	0.31
		Binary	876	62 (7.1)	16 (1.8)	14 (87.5)	2.9 (1.0)		
		Nonbinary	537	169 (31.5)	11 (2.0)	11 (100.0)	3.4 (1.4)		
	Double mastectomy	All	1413	647 (45.8)	637 (45.1)	637 (100.0)	4.4 (1.0)	1.7 (1)	0.19
		Binary	876	361 (41.2)	477 (54.5)	477 (100.0)	4.3 (1.0)		
		Nonbinary	537	286 (53.3)	160 (29.8)	160 (100.0)	4.4 (0.9)		
	Facial masculinization	All	1413	179 (12.7)	NA	NA	NA	NA	NA
		Binary	876	176 (20.1)	NA	NA	NA		
		Nonbinary	537	3 (0.6)	NA	NA	NA		
	Masculinizing body contouring	All	1413	589 (41.7)	25 (1.8)	23 (92.0)	4.0 (1.0)	0.2 (1)	0.66
		Binary	876	390 (44.5)	19 (2.2)	17 (89.5)	4.1 (1.0)		
		Nonbinary	537	199 (37.1)	6 (1.1)	6 (100.0)	3.8 (1.2)		
	Metoidioplasty	All	1413	675 (47.8)	10 (0.7)	9 (90.0)	3.8 (1.0)	0.0 (1)	0.84
		Binary	876	534 (61.0)	9 (1.0)	8 (88.9)	3.8 (1.0)		
		Nonbinary	537	141 (26.3)	1 (0.2)	1 (100.0)	4.0 (NA)		
	Oophorectomy	All	1413	845 (59.8)	144 (10.2)	143 (99.3)	4.6 (1.0)	7.1 (1)	0.008
		Binary	876	584 (66.7)	122 (13.9)	121 (99.2)	4.6 (0.9)		
		Nonbinary	537	261 (48.6)	22 (4.1)	22 (100.0)	4.1 (1.3)		
	Phalloplasty	All	1413	541 (38.3)	10 (0.7)	8 (80.0)	4.3 (1.0)		

© 2025 Pletta DR et al. *JAMA Network Open*.

Gender Identity	Surgery	Binary Status	N	Surgery Status: Desired but Unmet, No. (%)	Surgery Status: Received, No. (%)	N with Satisfaction Data, No. (%)	Procedural Satisfaction, Mean (SD)	*H* (df)	p-value
		Binary	876	459 (52.4)	10 (1.1)	8 (80.0)	4.3 (1.0)	NA	NA
		Nonbinary	537	82 (15.3)	NA	NA	NA		
	Removal of cervix	All	1413	703 (49.8)	150 (10.6)	149 (99.3)	4.6 (0.9)	0.5 (1)	0.46
		Binary	876	511 (58.3)	118 (13.5)	117 (99.2)	4.6 (0.9)		
		Nonbinary	537	192 (35.8)	32 (6.0)	32 (100.0)	4.6 (0.9)		
	Removal of uterus	All	1413	868 (61.4)	161 (11.4)	160 (99.4)	4.7 (0.9)	1.0 (1)	0.33
		Binary	876	586 (66.9)	126 (14.4)	125 (99.2)	4.7 (0.9)		
		Nonbinary	537	282 (52.5)	35 (6.5)	35 (100.0)	4.5 (1.1)		
	Scrotoplasty	All	1413	580 (41.0)	12 (0.8)	10 (83.3)	4.2 (0.8)	NA	NA
		Binary	876	508 (58.0)	12 (1.4)	10 (83.3)	4.2 (0.8)		
		Nonbinary	537	72 (13.4)	NA	NA	NA		
	Vaginectomy	All	1413	471 (33.3)	19 (1.3)	19 (100.0)	4.8 (0.7)	NA	NA
		Binary	876	408 (46.6)	19 (2.2)	19 (100.0)	4.8 (0.7)		
		Nonbinary	537	63 (11.7)	NA	NA	NA		
	Vocal cord/Voice surgery	All	1413	107 (7.6)	1 (0.1)	1 (100.0)	2.0 (NA)	1.2 (1)	0.27
		Binary	876	81 (9.2)	1 (0.1)	1 (100.0)	2.0 (NA)		
		Nonbinary	537	26 (4.8)	NA	NA	NA		
Transfeminine	Breast implants	All	763	430 (56.4)	74 (9.7)	74 (100.0)	4.0 (1.3)	1.5 (1)	0.22
		Binary	628	376 (59.9)	68 (10.8)	68 (100.0)	4.0 (1.3)		
		Nonbinary	135	54 (40.0)	6 (4.4)	6 (100.0)	3.7 (1.0)		
	Butt augmentation	All	763	272 (35.6)	12 (1.6)	10 (83.3)	3.4 (1.2)	NA	NA
		Binary	628	229 (36.5)	11 (1.8)	10 (90.9)	3.4 (1.2)		
		Nonbinary	135	43 (31.9)	1 (0.7)	0	NA		
	Facial feminization	All	763	516 (67.6)	52 (6.8)	52 (100.0)	4.1 (1.2)	0.4 (1)	0.51
		Binary	628	445 (70.9)	47 (7.5)	47 (100.0)	4.1 (1.3)		

© 2025 Pletta DR et al. *JAMA Network Open*.

Gender Identity	Surgery	Binary Status	N	Surgery Status: Desired but Unmet, No. (%)	Surgery Status: Received, No. (%)	N with Satisfaction Data, No. (%)	Procedural Satisfaction, Mean (SD)	*H* (df)	p-value
		Nonbinary	135	71 (52.6)	5 (3.7)	5 (100.0)	4.6 (0.5)		
	Feminizing body contouring	All	763	342 (44.8)	13 (1.7)	13 (100.0)	3.5 (1.1)	0.5 (1)	0.49
		Binary	628	290 (46.2)	12 (1.9)	12 (100.0)	3.5 (1.2)		
		Nonbinary	135	52 (38.5)	1 (0.7)	1 (100.0)	3.0 (NA)		
	Labiaplasty	All	763	466 (61.1)	82 (10.7)	82 (100.0)	4.0 (1.2)	0.4 (1)	0.52
		Binary	628	403 (64.2)	73 (11.6)	73 (100.0)	4.0 (1.2)		
		Nonbinary	135	63 (46.7)	9 (6.7)	9 (100.0)	3.7 (1.4)		
	Orchiectomy	All	763	464 (60.8)	118 (15.5)	117 (99.2)	4.5 (1.1)	0.1 (1)	0.71
		Binary	628	390 (62.1)	104 (16.6)	103 (99.0)	4.5 (1.1)		
		Nonbinary	135	74 (54.8)	14 (10.4)	14 (100.0)	4.5 (1.1)		
	Tracheal shave	All	763	382 (50.1)	39 (5.1)	39 (100.0)	3.9 (1.2)	1.7 (1)	0.19
		Binary	628	331 (52.7)	34 (5.4)	34 (100.0)	3.8 (1.3)		
		Nonbinary	135	51 (37.8)	5 (3.7)	5 (100.0)	4.6 (0.5)		
	Vaginoplasty	All	763	465 (60.9)	95 (12.5)	95 (100.0)	4.0 (1.3)	0.4 (1)	0.53
		Binary	628	405 (64.5)	84 (13.4)	84 (100.0)	4.1 (1.2)		
		Nonbinary	135	60 (44.4)	11 (8.1)	11 (100.0)	3.6 (1.6)		
	Vocal cord/Voice surgery	All	763	344 (45.1)	4 (0.5)	4 (100.0)	3.3 (1.7)	0.0 (1)	0.99
		Binary	628	305 (48.6)	4 (0.6)	4 (100.0)	3.3 (1.7)		
		Nonbinary	135	39 (28.9)	NA	NA	NA		

Abbreviations. SD, standard deviation; *H*, Kruskal-Wallis test statistic; df, degrees of freedom; NA, not available.

Note. Kruskal-Wallis tests were used to assess statistically significant differences in mean satisfaction scores between binary and nonbinary patients within the transmasculine and transfeminine strata. Statistical significance was established at α=0.05.

[a] Denominator is total stratum N.

© 2025 Pletta DR et al. *JAMA Network Open*.

[b] Denominator is the total number of patients within the stratum that reported receiving the procedure.

© 2025 Pletta DR et al. *JAMA Network Open*.

eTable 2. Encountered Barriers to Gender-Affirming Surgery (N = 2176)

Barrier	No. (%)
Experienced any barrier to gender-affirming surgery	2054 (94.4)
Cost of surgeries/procedures	1455 (66.9)
Worried about complications from medical procedures	992 (45.6)
Worries about short-term post-operative care (e.g., using a catheter, drains, wound care)	787 (36.2)
Cost of co-pays or co-insurance to see a medical provider/therapist	772 (35.5)
Unable to take time off from work	570 (26.1)
Long waiting time or wait list(s) to see a health care provider	544 (25.0)
Another barrier to surgery[a]	494 (22.7)
Not having someone to help care for me	485 (22.3)
Worried about long-term post-operative care (e.g., dilation)	398 (18.3)
No health coverage/insurance	350 (16.1)
Cost of prescriptions	297 (13.7)
Not able to work	294 (13.5)
Mistreatment by providers in the past	281 (12.9)
Transportation	264 (12.1)
Unable to find gender-affirming health care providers (e.g., providers/therapists who use correct name and pronouns)	199 (9.2)
Mistreatment by staff and/or other patients for being transgender (does not include providers)	183 (8.4)
Inconvenient hours for appointments	164 (7.5)
Worried about safety getting to/from the healthcare provider	136 (6.3)
No safe and clean place to stay after surgery	104 (4.8)
Worried about being seen at a clinic by friends, family members, or acquaintances	103 (4.7)
Childcare	34 (1.6)

Note. Barriers are listed descending from most commonly to least commonly reported. Percentages were calculated using the total sample (N=2176).

[a] Another barrier to surgery included heterogeneous write-in responses such as: “fatphobia”, “COVID-19 putting a halt to ‘elective’ surgeries”, “not taken seriously and therefore slowed down in the process”, “going away for a semester and

© 2025 Pletta DR et al. *JAMA Network Open*.

unable to start before then”, “denied bottom surgery due to my weight.”, “[institution] canceled my surgery without giving me a reason”.

© 2025 Pletta DR et al. *JAMA Network Open*.
