# Supplementary material for: Gender-Affirming Surgical History, Satisfaction, and Unmet Needs Among Transgender Adults
Source: JAMA Netw Open. 2025 Sep 18;8(9):e2532494. doi: 10.1001/jamanetworkopen.2025.32494 (PMC12447237; doi:10.1001/jamanetworkopen.2025.32494)
Supplement: Supplement 2. — Data Sharing Statement [file jamanetwopen-e2532494-s002.pdf]

Data Sharing Statement

Pletta. Gender-Affirming Surgical History, Satisfaction, and Unmet Needs Among Transgender Adults. *JAMA Netw Open*. Published September 18, 2025.
doi:10.1001/jamanetworkopen.2025.32494

Data
Data available: No

Additional Information
Explanation for why data not available: Data from this study are not publicly available for use. For data requests, please email the corresponding author. Data requests will be considered from investigators with funding support, after approval of a concept proposal by all study investigators, and with a signed data access agreement.
